# Functionally declining microbiota in chronic disease: beyond taxonomic loss

**DOI:** 10.1080/19490976.2026.2748134

**Published:** 2026-10-15

**Authors:** Heenam Stanley Kim

**Affiliations:** a Division of Biosystems & Biomedical Sciences, College of Health Sciences, Korea University, Seoul, Korea

**Keywords:** Gut microbiota, functional decline, functionally declining microbiota, disappearing microbiota, taxonomic loss

## Abstract

Microbiota deterioration in modern human populations has been linked to chronic disease, and taxonomic profiling is increasingly used to understand, diagnose, and guide treatment. Yet such profiles do not fully capture disease-relevant microbiota states. I propose functional decline within retained taxa as a distinct axis of microbiota deterioration, complementary to taxonomic loss. Modern environmental pressures can drive not only taxonomic loss but also directional selection toward variants or lineages with reduced host-beneficial function or increased harmful activity. Two mechanistically characterized systems from studies by my group illustrate this process: antibiotic-selected *Akkermansia* variants with diminished metabolic benefits, and *Faecalibacterium* pathobionts that promote disease when dominant over beneficial strains, with supporting evidence from atopic dermatitis and pediatric inflammatory bowel disease. Evidence from additional gut taxa suggests that this pattern may extend beyond these two examples. By integrating this hidden axis of functional decline with the well-established axis of taxonomic loss, the proposed two-axis framework could help clarify inc onsistent microbiota–disease associations and guide strain-resolved, function-first approaches to microbiota analysis and intervention.

## Graphical abstract

**Figure:**
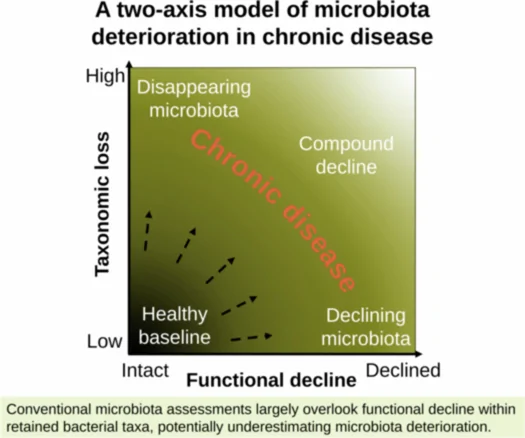

## Introduction

The human gut microbiota is changing rapidly across industrialized populations, where community structures differ markedly from those of traditional societies and have been linked to chronic disease.^1-3^ The central question is not simply whether the microbiota has changed, but which aspects of this deterioration contribute to disease.

The dominant explanation, articulated by Martin Blaser, is the “disappearing microbiota” hypothesis.^1^ This model proposes that industrialization-associated pressures—including antibiotic exposure, disrupted early-life microbial transmission, and dietary change—drive the progressive loss of microbial species that co-evolved with humans. The resulting depletion of key taxa is thought to erode essential ecological and immunological functions, thereby increasing susceptibility to disease. This perspective has shaped much of modern microbiota research, motivating efforts to catalog microbial diversity and link its disruption to disease risk.^4^

Yet taxonomic composition alone often fails to distinguish healthy from diseased states.^5^ ^,^ ^6^ Moreover, efforts to define a shared ancestral or “core” human microbiota have yielded divergent and poorly reproducible candidates.^7-9^ Although part of this difficulty is methodological and definitional,^9^ the lack of reproducibility contrasts with findings from non-human systems, in which microbiota profiling has revealed reproducible, host-specific, or phylogenetically structured communities, including in social insects,^10^ ^,^ ^11^ great apes,^12^ and other mammals,^13^ with maternal or colony-level transmission shown to contribute to their maintenance in some cases.^12-14^ Although these systems differ from human populations in many respects and therefore may not be directly comparable, they suggest that host-associated microbiotas can maintain reproducible taxonomic structure under relatively stable ecological and transmission conditions.

One possible explanation for the lack of reproducible taxonomic structure in industrialized human microbiotas, in contrast to that observed in non-human systems, is that decades of industrialization-associated pressures may have produced cumulative deterioration, increasing interindividual variability and obscuring a definable “core” microbiota. Taxonomic profiling may capture only part of this deterioration, with taxonomic loss representing the visible compositional component of a broader underlying decline.

This manuscript develops a framework in which functional decline within retained taxa represents a second, distinct axis of microbiota deterioration, complementary to taxonomic loss. The proposal grows primarily from two lines of work from my laboratory: studies of antibiotic-selected *Akkermansia* variants that remain present while losing host-beneficial capacity,^15-17^ and studies of *Faecalibacterium* lineage imbalance in atopic dermatitis, in which a (potential) pathobiont (a normally commensal member of the microbiota that can promote disease under particular host or ecological conditions) lineage expands at the expense of a beneficial lineage.^18^ ^,^ ^19^ Notably, the latter ecological pattern is supported by independent evidence from pediatric inflammatory bowel disease, where Zhang et al. reported a comparable shift among *Faecalibacterium* lineages.^20^ Whereas the earlier studies focused on specific manifestations of functional deterioration, the present manuscript generalizes these observations across taxa and mechanisms. The advance proposed here is to recognize them not as unrelated findings, but as examples of a broader mode of microbiota deterioration arising through distinct mechanisms—genetic change in one case and ecological selection in the other—and to integrate this mode with taxonomic loss into a two-axis framework. This framework may provide a more complete account of disease-associated microbiota dysfunction than taxonomic approaches alone.

## Functional decline within the microbiota—alongside taxonomic loss

The second axis of microbiota decline is what I term the functionally declining microbiota: functional deterioration within taxa that remain detectable. Environmental pressures can favor genetic variants or lineages with reduced host-beneficial function or increased harmful activity, allowing them to expand within the retained taxon. Functional decline therefore reflects a directional, exposure-driven change in population function rather than static strain heterogeneity (Box 1).

Box 1.
What distinguishes functional decline within retained taxa from ordinary strain variation?

Strain variation is a normal feature of the microbiota. It becomes functional decline when environmental pressure drives a directional population shift that alters the net function of a retained taxon. A strong case would ideally satisfy all four criteria below.

1. **Environmental pressure.** An identifiable environmental exposure, such as antibiotic exposure, dietary change, or inflammation, that selects for a new genetic variant or favors a pre-existing variant or lineage. The favored population exhibits increased survival or growth under that condition.
2. **Directional shift.** The favored population expands over time or displaces beneficial populations, rather than merely coexisting stably with them.
3. **Functional consequence at the taxon level.** The favored population exhibits reduced or lost host-beneficial function, increased harmful activity, or both, and becomes sufficiently abundant for these altered properties to reduce the net host-beneficial function of the taxon, increase its net harmful activity, or both.
4. **Altered host phenotype.** The resulting taxon-level functional shift is associated with, and ideally shown to contribute to, an adverse host phenotype or disease-relevant outcome.

Functional decline should be distinguished from context-dependent effects of an otherwise unchanged microbial population. A given strain may exert different host effects under different host or environmental conditions without being functionally declined. Accordingly, the functional decline axis should be interpreted within a defined host and environmental context, with decline assessed relative to an appropriate reference or ancestral state under the same conditions.

## Genetic change followed by ecological selection: the *Akkermansia* paradigm

*Akkermansia* illustrates how genetic change can produce variants that are subsequently selected and enriched under environmental pressure (Figure 1). Antibiotic exposure can select for mutants with enhanced survival and promote their enrichment. Importantly, this survival advantage may come at the cost of diminished host-beneficial function (Box 1).

**Figure 1.**
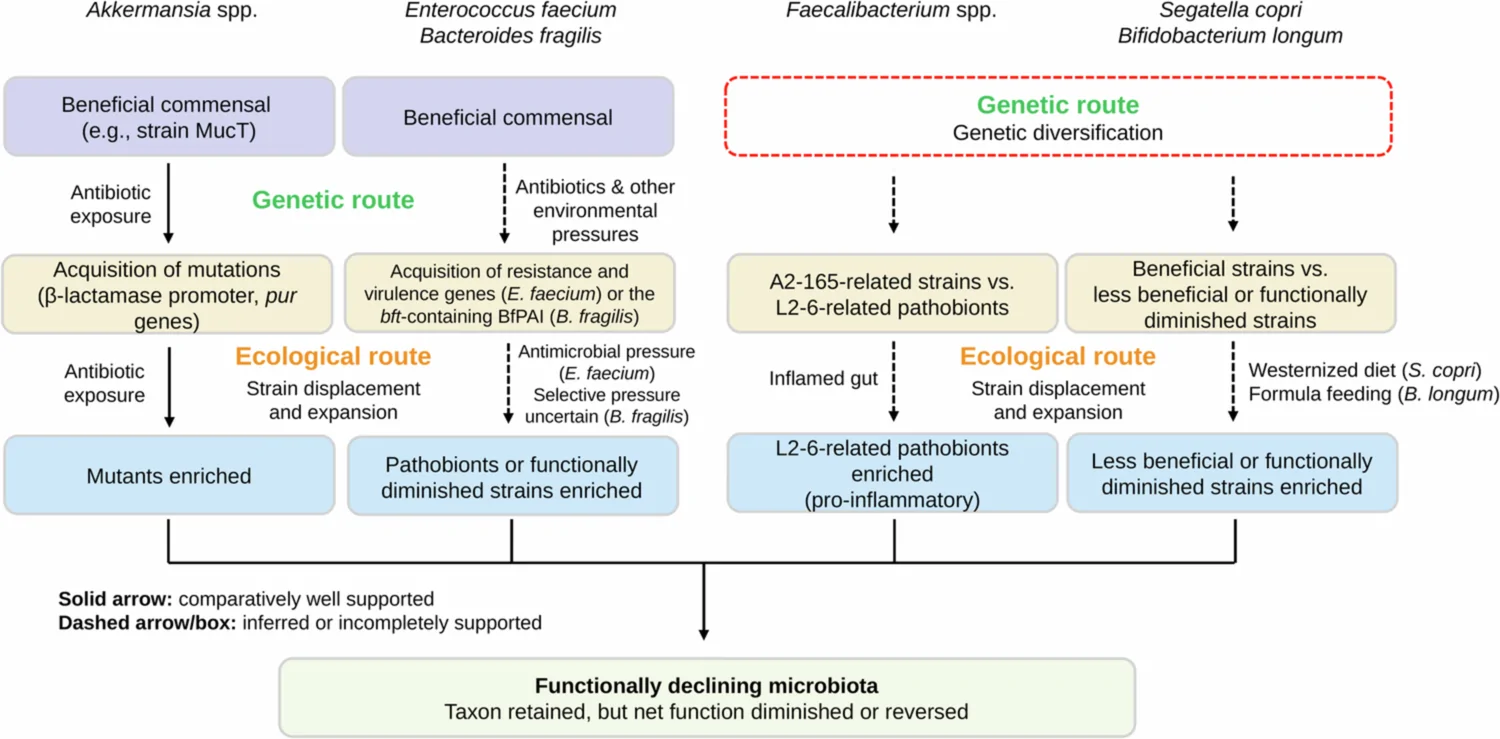
Genetic and ecological routes to directional functional decline within retained taxa. Left, antibiotic exposure selects *Akkermansia* variants carrying *β*-lactamase promoter or *pur* mutations. These variants survive antibiotic exposure but provide diminished metabolic benefits and may become enriched under continued selection. In *Enterococcus faecium*, sustained antimicrobial and other hospital-associated pressures have selected hospital-adapted lineages carrying acquired resistance and virulence genes. In *Bacteroides fragilis*, acquisition of the *bft*-containing pathogenicity island (BfPAI) gives rise to enterotoxigenic strains with inflammatory and tumor-promoting activity, although the pressures driving their directional enrichment remain uncertain. Right, in *Faecalibacterium*, inflammatory conditions may favor pathobiont lineages, such as L2-6-related/*F. longum*, over beneficial A2-165-related/*F. duncaniae*, shifting the taxon’s net activity toward a more pro-inflammatory state. In *Segatella copri* and *Bifidobacterium longum*, Westernized diets and formula feeding may favor less beneficial *S. copri* clades and less HMO-adapted *B. longum* populations, respectively. The proposed ecological route presupposes antecedent genetic diversification that generated the functionally distinct lineages subsequently subject to selection. Solid arrows indicate comparatively well-supported steps, whereas dashed arrows and the dashed box indicate inferred or incompletely established steps, including antecedent genetic diversification and uncertain selective pressures. Across taxa, these processes converge on a declining microbiota, in which a taxon remains detectable but its net host-beneficial function is diminished or reversed.

*Akkermansia muciniphila* is a mucin-degrading bacterium that resides in the colonic mucus layer and is strongly associated with metabolic health.^21^ Across human cohorts and animal models, *A. muciniphila* abundance inversely correlates with obesity, type 2 diabetes, and metabolic syndrome.^22-24^ In mice, administration of *A. muciniphila* reduces weight gain, improves insulin sensitivity, and strengthens gut barrier integrity.^22^ ^,^ ^25^ These findings have established *A. muciniphila* as a leading next-generation probiotic candidate.^25^

Against this background, Han et al. showed that the beneficial effects of *A. muciniphila* can be lost even when the species remains present.^15^ In that study, a human-derived *A. muciniphila* isolate was subjected experimentally to lethal penicillin exposure in vitro, and surviving variants were isolated and genome-sequenced. Among nine selected variants, mutations occurred either in the promoter of a TEM-type *β*-lactamase gene or in *pur* genes involved in de novo purine biosynthesis; representative variants were subsequently tested for their host effects in a mouse model. Related mutational signatures were then identified in publicly available *A. muciniphila* genome sequences from geographically diverse human-derived strains, indicating that comparable variants occur in human populations, although their evolutionary histories and selective exposures in those populations cannot be determined from these sequence data alone.

Critically, these variants showed impaired host-beneficial function.^15^ When gavaged into mice fed an obesogenic diet, mutant strains failed to reduce fat gain, improve glucose tolerance, or strengthen gut barrier integrity—protective effects observed with wild-type strains.^15^ Antibiotics may therefore act as selective filters, enriching strains that survive exposure but lose host-beneficial capacity.^15^ ^,^ ^16^ This mechanism could help explain inconsistent associations between *A. muciniphila* abundance and metabolic health,^26-28^ as well as the reported “duality” of *A. muciniphila*,^29^ which may reflect the presence of functionally distinct strains that species-level surveys cannot distinguish. Notably, the variants identified by Han et al. appear geographically widespread and may persist through vertical or interpersonal transmission, potentially increasing their global influence.^15^ ^,^ ^30^

Together, these findings support a conceptual selection-amplification process (Figure 1): antibiotic exposure selects for and begins to enrich variants with a relative survival advantage but compromised host-beneficial capacity, and subsequent exposures may amplify their enrichment within host-associated communities. Although these variants are globally widespread and show evidence consistent with transmission between individuals and across generations, direct evidence that they expand at the expense of wild-type, host-beneficial strains in human populations remains limited. Nevertheless, the key clinical problem may lie not only in the antibiotic-mediated depletion of *A. muciniphila*, but also in the persistence and expansion of variants with diminished host-beneficial activity.^17^

## Ecological enrichment of pathobiont lineages: the *Faecalibacterium* paradigm

Functional decline can also occur when environmental conditions favor pathobiont lineages over closely related beneficial competitors occupying the same niche. The *Faecalibacterium* case illustrates this ecological enrichment route (Figure 1).

*Faecalibacterium prausnitzii* has historically been treated as a health-associated species.^31^ Its abundance is frequently reduced in inflammatory bowel disease (IBD), and its metabolites—particularly butyrate—support epithelial barrier integrity, promote regulatory T cell differentiation, and suppress pro-inflammatory cytokine production.^32-35^

Yet *F. prausnitzii* presents a paradox similar to that of *A. muciniphila*: its abundance does not consistently predict disease status. Although many studies report depletion in Crohn’s disease, ulcerative colitis, and metabolic disorders, others report enrichment, including in pediatric Crohn’s disease,^36^ atopic dermatitis (AD),^18^ and childhood obesity.^37^

In AD, Song et al. addressed this paradox through strain-level analysis of *F. prausnitzii* populations in children.^18^ The populations were functionally heterogeneous. A phylogenetically distinct lineage related to strain L2-6, likely functioning as a pathobiont, was enriched in patients with AD, whereas beneficial strains related to A2-165 were depleted (Figure 1). This shift was accompanied by reduced fecal butyrate and propionate levels and increased metagenomic signatures of auxotrophic and opportunistic bacteria, consistent with an altered, inflammation-associated gut environment.

A similar lineage shift was independently observed beyond AD. Zhang et al. applied strain-resolved metaproteomics to a treatment-naïve pediatric IBD cohort.^20^ At the mucosal–luminal interface, the L2-6-related *F. prausnitzii* lineage was dominant and enriched in Crohn’s disease, whereas the A2-165-related lineage was depleted—paralleling the L2-6/A2-165 imbalance described by Song et al. in AD. Zhang et al. proposed that oxidative stress in the inflamed gut may favor expansion of the L2-6 lineage, which encodes distinctive oxidative-stress-response functions. Although the drivers of pathobiont enrichment remain incompletely defined, inflammatory gut conditions may therefore create selective environments that favor pathobiont expansion.^18^ ^,^ ^20^ The recurrence of this imbalance across distinct diseases, together with a plausible selective mechanism, supports its interpretation as a reproducible ecological redistribution rather than a purely cohort- or disease-specific association.

Strains L2-6 and A2-165 are now classified as *F. longum* and *F. duncaniae*, respectively,^38^ ^,^ ^39^ underscoring the genomic divergence between these lineages. This reclassification confirms that populations previously grouped within *F. prausnitzii* are genomically distinct. Together with evidence of their contrasting functional properties, it further illustrates why measurements that pool these populations may obscure a disease-relevant redistribution between beneficial and pathobiont lineages.

The causal relevance of this disease-associated lineage-enrichment model is further supported by Lee et al.^19^, who showed that human-derived *Faecalibacterium* pathobionts can promote AD-like phenotypes in mice and transmit disease susceptibility from mothers to offspring. Maternal administration of pathobiont strains induced AD-like phenotypes in offspring, and these effects were amplified by a maternal low-fiber diet. Conversely, administration of the beneficial A2-165 strain counteracted these effects. These findings experimentally link lineage composition to host phenotype and support ecological enrichment of pathobiont lineages as a causal route by which functional decline can alter host physiology, although validation of this mechanism in humans remains necessary.

While inflammatory conditions may favor *Faecalibacterium* pathobionts,^18^ ^,^ ^20^ these pathobionts can in turn promote intestinal inflammation, as demonstrated by Lee et al.^19^ This reciprocal relationship may create a feed-forward loop in which inflammation promotes pathobiont enrichment, which then further reinforces inflammation. Industrialization-associated pressures, including dietary change and antibiotic exposure, may initiate this process by creating conditions that favor an initial lineage shift, after which the cycle may become self-reinforcing.

The ecological shift described here acts on pre-existing genetic and functional diversification within *Faecalibacterium*, which has produced lineages ranging from beneficial commensals to pathobionts (Figure 1). The evolutionary processes underlying this diversification remain poorly understood.

## Beyond the mechanistic core: broader evidence for functional decline

The *Akkermansia* and *Faecalibacterium* cases provide mechanistic evidence for functional decline and its consequences for host health. Additional taxa provide broader support for this model. Some show pressure-driven divergence toward clinically harmful lineages, whereas others demonstrate the existence of opposing functional states or suggest exposure-associated selection among lineages with differing host effects. Together, these examples indicate that key components of functional decline recur across diverse gut taxa, selective pressures, and life stages. However, they vary in how fully they satisfy the defining criteria (Box 1), and much of the evidence for directional selection and host consequences remains indirect or awaits validation in longitudinal human studies.

### Enterococcus faecium: functional divergence through pressure-driven gene acquisition

*Enterococcus faecium* provides a possible example of antimicrobial-driven functional divergence within a retained species, with direct clinical consequences (Figure 1). Some *E. faecium* strains have been used as probiotics, with reported benefits including pathogen inhibition, metabolic contributions, and immunomodulatory effects.^40^ By contrast, other strains belong to the hospital-adapted clade A1, which is distinct from commensal populations.^41^ ^,^ ^42^

Hospital-adapted *E. faecium* was shaped by antimicrobial pressure, with gene acquisition contributing substantially to its divergence.^42^ ^,^ ^43^ Its acquired repertoire includes antibiotic-resistance determinants, such as genes conferring vancomycin resistance (*vanA*/*vanB*) and ampicillin resistance, as well as colonization- and virulence-associated factors, including the *esp* pathogenicity island and pilus clusters.^42^ Antimicrobial selection has therefore favored lineages with enhanced capacity to survive, colonize, and cause infection in healthcare settings. This process parallels the selection of survival-enhancing chromosomal mutations in *Akkermansia* under antibiotic pressure: in both systems, antimicrobial exposure favored populations with enhanced survival, but with different functional consequences. Antibiotic-selected *Akkermansia* variants lost host-protective capacity, whereas hospital-adapted *E. faecium* lineages acquired traits promoting colonization and pathogenicity.

The consequences of this divergence are clinically important. When microbiota-mediated colonization resistance is reduced, as can occur after antibiotic exposure, hospital-adapted *E. faecium* can expand under continued antimicrobial selection, translocate across the epithelial barrier, and cause bloodstream infection, particularly in immunocompromised individuals such as hematopoietic stem-cell transplant recipients and other high-risk patients.^44^ ^,^ ^45^ Thus, *E. faecium* provides an additional example of how antimicrobial pressure can favor survival-enhanced lineages with increased harmful potential. However, the evolutionary steps by which clade A1 emerged from commensal populations and the contributions of individual acquired traits to its pathogenic behavior remain incompletely defined.

### Bacteroides fragilis: opposing functional states within a retained species

*Bacteroides fragilis* provides an additional example of functional diversification within a retained species, associated largely with the acquisition of genetic elements that have consequences for host health (Figure 1). Many non-toxigenic *B. fragilis* (NTBF) strains produce polysaccharide A (PSA), a zwitterionic capsular polysaccharide that promotes IL-10-producing regulatory T cells through Toll-like receptor 2 signaling.^46^ ^,^ ^47^ PSA-mediated immune modulation protects against experimental colitis, supports host–commensal mutualism, and restores immune balance in germ-free models.^46^ ^,^ ^48^ Accordingly, PSA-producing NTBF strains have long served as a model system for beneficial host–commensal immune modulation.

By contrast, enterotoxigenic *B. fragilis* (ETBF) strains carry the *bft* gene, which encodes fragilysin, a metalloprotease that disrupts epithelial barrier integrity and activates NF-κB-dependent inflammatory signaling.^49^ ^,^ ^50^ The *bft* gene resides within the approximately 6-kb *B. fragilis* pathogenicity island (BfPAI), which is itself carried on the larger putative conjugative transposon CTn86, consistent with horizontal acquisition of toxigenic capacity ^51^ ^,^ ^52^—a genetic route that parallels the acquisition of resistance and virulence determinants in *E. faecium*. ETBF colonization has been causally linked to intestinal inflammation and accelerated colorectal tumorigenesis in mice,^53^ ^,^ ^54^ and ETBF has been enriched in mucosal biofilms from patients with colorectal cancer.^55^ ^,^ ^56^

This distinction is significant because ETBF represents a pathobiont state within a species that also contains beneficial commensal strains. However, *B. fragilis* remains only a partial example of functional decline because there is limited evidence that contemporary environmental pressures selectively enrich ETBF or drive a directional population shift from NTBF toward ETBF.

### Segatella copri: potential diet-associated lineage selection

*Segatella copri* (formerly *Prevotella copri*) illustrates a potential route of diet-driven ecological selection within a retained taxon (Figure 1). *S. copri* is enriched in many non-industrialized populations consuming fiber-rich diets, and its carbohydrate-fermenting activity has been associated with favorable metabolic outcomes, including improved glucose tolerance.^57-59^ In other cohorts, however, *S. copri* has been associated with inflammatory and metabolic disease states, including rheumatoid arthritis, insulin resistance, and systemic inflammation.^60^ ^,^ ^61^ These contrasting associations have complicated interpretation of its role in human health.

Metagenomic studies have provided a possible explanation by showing that *S. copri* comprises multiple phylogenomic clades and strains with distinct metabolic capacities.^62^ Non-industrialized populations harbor greater clade diversity, often with multiple clades coexisting within individuals, whereas this diversity is reduced in industrialized populations. Populations associated with fiber-rich diets exhibit greater genetic potential for complex-carbohydrate degradation. By contrast, strains associated with Westernized diets may exhibit less favorable metabolic potential, including a higher prevalence of genes involved in branched-chain amino acid biosynthesis and a reduced genetic capacity for complex-carbohydrate degradation.^58^ ^,^ ^63^

Together, these observations suggest that diet may shape the relative abundance and distribution of *S. copri* lineages, with fiber-rich diets preferentially supporting carbohydrate-degrading strains and Westernized diets potentially favoring strains with less favorable metabolic potential.^58^ ^,^ ^64^ Alongside the *Faecalibacterium* case, *S. copri* represents another potential example of an ecological route to functional decline, in which diet shifts the balance among pre-existing, functionally divergent lineages. However, direct evidence that dietary Westernization drives the depletion of host-beneficial *S. copri* lineages or the expansion of harmful lineages with demonstrable adverse effects on the host remains limited.

### Bifidobacterium longum: early-life selection of functional capacity

*B. longum* provides an early-life example of potential functional decline associated with limited HMO availability during formula feeding (Figure 1). As an early colonizer of the infant gut, *B. longum* plays an important role in shaping the founding gut ecology and influencing infant health.^65^ ^,^ ^66^ Yet its strains differ markedly in the functions they provide.

Breastfed infants are often colonized by HMO-adapted *B. longum* populations, including *B. longum* subsp. *infantis*, which carries an extensive repertoire of HMO transporters and intracellular HMO-degrading enzymes.^67^ ^,^ ^68^ These strains ferment HMOs into acetate and lactate, lowering colonic pH and thereby suppressing opportunistic pathogens, including members of the Enterobacteriaceae.^65^ ^,^ ^69^ They also produce indole-3-lactic acid, an AhR/HCA3 agonist with immunomodulatory and anti-inflammatory effects in the developing intestine.^70^ ^,^ ^71^ By contrast, formula-fed infants more commonly harbor *B. longum* subsp*. longum*, which carries a broader glycoside hydrolase repertoire suited to diverse carbohydrates but generally has more limited HMO-utilization capacity.^68^ Consequently, these populations may provide less of the HMO-dependent ecological and immunological activity associated with HMO-adapted strains.

These differences may be particularly consequential during initial colonization, when maternally transmitted *Bifidobacterium* strains help establish the infant gut ecology.^72^ *B. longum* thus illustrates a plausible mechanism by which formula feeding could favor a functionally different population structure: as in the *Faecalibacterium* case, decline would occur not through loss of the species but through a shift among functionally distinct populations retained within it. Although breastfeeding is consistently associated with reduced infection risk and lower odds of later overweight, obesity, and type 2 diabetes,^73^ ^,^ ^74^ these observational associations may also be influenced by residual confounding. The extent to which HMO-adapted *B. longum* contributes causally to these effects—and, conversely, whether its lower representation in formula-fed infants contributes to adverse outcomes—therefore remains uncertain. Direct evidence that reduced HMO availability drives a directional and persistent population shift sufficient to reduce net host-beneficial function also remains limited.

Taken together, these six taxa vary substantially in how fully they satisfy the criteria for functional decline defined in Box 1. The type and the extent of strength and of evidence supporting each criterion are summarized in Table 1.

**Table 1.** Evidence supporting the Box 1 criteria across six representative taxa. Cells indicate the available supporting evidence types: HX, human cross-sectional; HL, human longitudinal; AC, animal causal; IV, in vitro experimental; —, absent or not established.

Taxon	1. Environmental pressure	2. Directional shift	3. Functional consequence at the taxon level	4. Altered host phenotype
***A. muciniphila***	Antibiotic exposure selects *β*-lactamase promoter and *pur* variants (IV; 15)	Selection and enrichment of antibiotic-adapted variants (IV; 15)	Variants exhibit reduced host-beneficial function (AC; 15)	Loss of protection against metabolic dysfunction in mice (AC; 15)
***Faecalibacterium spp.***	Inflammatory conditions may favor pathobiont lineages (HX; 18, 20); low-fiber diet acts synergistically with pathobiont enrichment to promote disease phenotypes (AC; 19)	Shift toward L2-6-related/*F. longum* lineages and away from A2-165-related/*F. duncaniae* lineages in two independent cohorts (HX; 18, 20)	Enrichment of pathobiont populations with reduced beneficial and/or increased harmful activity (HX; 18, 20; AC; 19)	AD-like phenotypes induced experimentally (AC; 19); disease-associated lineage shifts observed in childhood AD and IBD (HX; 18, 20)
***E. faecium***	Antimicrobial and hospital-associated pressures; acquisition of resistance and virulence determinants (HX; 41–43)	Enrichment/expansion of hospital-adapted lineages under antimicrobial selection (HX/HL; 41–45)	Increased colonization, resistance, and virulence potential (HX; 42, 43)	Intestinal domination preceding bloodstream infection (HL; 44, 45)
***B. fragilis***	Selective pressure not established; acquisition of the *bft*-containing pathogenicity island is documented (51, 52)	—	Acquisition of enterotoxigenic and pro-inflammatory activity (IV/AC; 49, 50, 53, 54)	Intestinal inflammation and accelerated colorectal tumorigenesis in mice (AC; 53, 54); enrichment in colorectal cancer-associated mucosa (HX; 55, 56)
***S. copri***	Diet-associated differences in lineage distribution and functional potential (HX; 58, 62–64)	Industrialized populations show altered clade distribution and reduced clade diversity, but direct longitudinal selection is not established (HX; 62–64)	Lineages differ in complex-carbohydrate utilization and other metabolic capacities (HX/IV; 58, 63)	Associations with rheumatoid arthritis, insulin resistance, and systemic inflammation (HX; 60, 61)
***B. longum***	Limited HMO availability/formula feeding associated with differences in strain composition (HL; 68)	Succession toward populations with differing substrate adaptations during early-life dietary change (HL; 68)	HMO-adapted and less HMO-adapted populations differ markedly in HMO utilization and metabolic output (IV/HL; 66–71)	—

## Unifying genetic and ecological routes to functional decline

Across these examples, a unifying framework emerges for the “declining microbiota,” in which retained taxa undergo directional shifts toward reduced host-beneficial capacity or increased harmful potential under industrialization-associated environmental pressures (Figure 1). This should be viewed as an operational framework for describing how such pressures may alter the functional composition of retained taxa, with taxon-specific details refined as evidence accumulates.

Mechanistically, genetic and ecological processes are analytically distinct but biologically coupled (Figure 1). Genetic processes generate variation through mutation, recombination, and gene acquisition, whereas environmental pressures determine which variants or lineages survive and expand. These processes need not occur as discrete stages: ecological selection may act on pre-existing variation, newly arising variants, or both. Conversely, altered or pathobiont-like lineages discussed primarily in ecological terms—including those within *Faecalibacterium* spp., *S. copri*, and *B. longum*—ultimately reflect prior genetic diversification that produced their contrasting functional capacities. Functional decline therefore reflects the population-level interaction between genetic variation and ecological selection and expansion, rather than either process alone.

Testing the declining microbiota framework will require appropriate cohorts or experimental systems, appropriate metadata and longitudinal structure where needed, and microbiome data with sufficient resolution or marker sets to distinguish functionally distinct bacterial lineages within the specific disease setting. For the *Akkermansia* model, for example, cohorts focused on metabolic disease would provide an appropriate setting to assess whether functionally altered variants are reproducibly associated with disease. For the *Faecalibacterium* model, longitudinal mother–infant cohorts would be essential for examining microbial transmission and subsequent disease development in offspring.

The framework is developed primarily from bacterial examples but is not inherently restricted to bacteria. Theoretically, analogous functional decline could occur in fungi, archaea, or viruses through genetic change or shifts among functionally distinct lineages within a taxon.

Importantly, functional decline can be missed by standard taxonomic profiling when analytical resolution is insufficient to distinguish functionally divergent populations. Several bacterial populations formerly grouped within *Faecalibacterium prausnitzii* and *Akkermansia muciniphila* have subsequently been reclassified into multiple genome-defined species.^75-77^ However, these genome-based divisions are not necessarily reflected in the 16S rRNA gene, so populations assigned to different species by whole-genome analysis may remain indistinguishable by partial 16S rRNA gene-based profiling. Species-aggregated shotgun metagenomic analyzes can likewise obscure functionally important variation below the species level. Strain-resolved approaches based on sequence variation, gene content, metagenome-assembled genomes, or targeted markers can in principle resolve such differences. However, focused analyzes may require prior knowledge of relevant strain-level variation or functionally divergent lineages. Thus, this blind spot reflects a mismatch between analytical resolution and the level at which functional divergence occurs, rather than an inherent limitation of microbiome analysis.

Because antibiotic exposure, low-fiber diets, and other industrialized lifestyle factors are widespread and increasingly global,^78^ ^,^ ^79^ this analytical blind spot could have broad relevance. If these pressures promote functional decline within persisting taxa, many microbiotas may appear taxonomically preserved while harboring functionally altered populations. Such changes could occur alongside—and potentially compound—the taxonomic loss already associated with industrialized lifestyles.

## A two-dimensional framework of microbiota deterioration

The declining microbiota concept, when integrated with the well-established disappearing microbiota framework,^1^ yields a two-dimensional model of microbiota decline (Figure 2). One axis captures taxonomic loss, whereas the other captures functional decline within retained taxa through directional shifts toward less beneficial or harmful variants or lineages, whether generated by mutation or gene acquisition or already present within the population.

**Figure 2.**
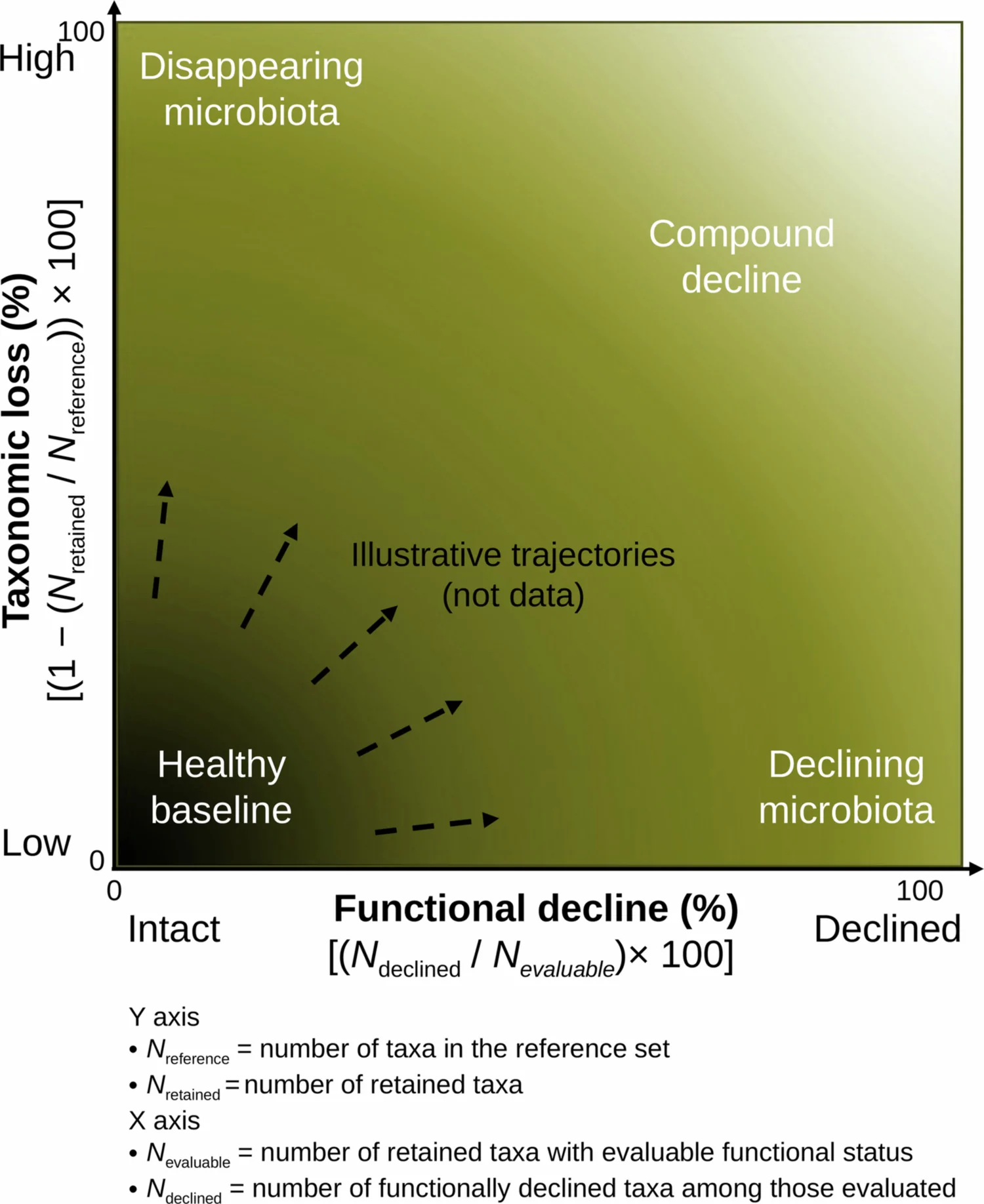
A two-dimensional framework of microbiota deterioration. Taxonomic loss forms one axis, whereas functional decline forms the other. As operational measures, taxonomic loss (%) can be expressed as the proportion of taxa lost relative to a defined reference set, whereas functional decline (%) can be expressed as the proportion of evaluable taxa classified as declined according to the Box 1 criteria, based on available strain-, lineage-, variant-, or function-level evidence. An initial panel may include retained taxa for which functional status can be evaluated and can be expanded as additional strain-level and functional data accumulate. Healthy baseline, disappearing microbiota, declining microbiota, and compound decline mark idealized corner anchors, whereas most microbiotas are expected to occupy intermediate positions within this continuous landscape. Dashed arrows illustrate potential trajectories away from a healthy baseline under pressures such as antibiotic exposure and dietary change, which may drive movement along either or both axes. The framework highlights a diagnostic blind spot: taxonomic profiles alone may underestimate the extent of microbiota deterioration by failing to capture functional decline within retained taxa.

To make this framework operational with available data, quantitative measures can be assigned to both axes. Taxonomic loss (Y axis) could be expressed as:

$$\mathrm{Taxonomic}\;\mathrm{loss}\;(\%)\;=\;(1\;-\;(N_{\mathrm{retained}}\;/\;N_{\mathrm{reference}}))\;\times \;100,$$

where *N*_reference_ is the number of taxa in the defined reference set, and *N*_retained_ is the number of those taxa that are retained. The reference set could be selected according to the study objective and derived from an appropriately matched healthy population or another prespecified baseline, such as a pre-exposure or earlier longitudinal time point, the union of taxa detected across the study cohort, a defined synthetic microbial community, or a well-characterized hunter-gatherer population.

Functional decline (X axis) could be expressed as:

$$\mathrm{Functional}\;\mathrm{decl}\mathrm{ine}\;(\%)=(N_{\mathrm{declined}}/N_{\mathrm{evaluable}})\times 100,$$

where *N*_evaluable_ is the total number of retained taxa for which functional status can be evaluated, and *N*_declined_ is the number of functionally declined taxa among those evaluated. The evaluable set could include *Akkermansia muciniphila*, *Faecalibacterium* spp., *Enterococcus faecium, Bacteroides fragilis, Segatella copri,* and *Bifidobacterium longum*, as represented in this manuscript. Declined taxa could be defined as those for which available evidence supports classification as functionally declined based on a prespecified application of the defining features listed in Box 1. Although functional decline may occur in many additional taxa and is likely to exist along a continuum within individual taxa, this limited scope and binary classification provide a practical starting point given the currently limited quantitative data and can be expanded and refined as additional information becomes available.

Together, these dimensions define a continuous landscape rather than discrete states (Figure 2). The corners of this landscape represent idealized anchors: a healthy baseline, disappearing microbiota, declining microbiota, and compound decline involving both processes. Most microbiotas are expected to occupy intermediate positions, reflecting varying degrees of taxonomic loss and functional decline.

However, most studies emphasize taxonomic loss while overlooking functional change within retained taxa. Thus, a microbiota may appear only mildly dysbiotic—or even relatively preserved based on taxonomic profiles—with key taxa still detectable and diversity remaining within expected ranges. However, it may be more severely functionally compromised than these profiles suggest if retained taxa are dominated by less beneficial or pathobiont-like variants. In this sense, dysbiosis assessed only through taxonomic composition may underestimate the true extent of microbiota deterioration when functional decline is also present.

Although the two axes are analytically distinct, they are biologically coupled because they unfold within the same ecological system. Antibiotic treatment, long-term dietary change, and other environmental pressures can drive taxonomic loss and functional decline simultaneously, while loss of niche competitors may further enable altered variants or lineages to expand. Conversely, functional decline may, under some conditions, reduce ecological fitness or stability and contribute to subsequent taxonomic loss. The framework therefore predicts that clinical phenotypes and intervention responses will be better explained by combined measures of taxonomic loss and functional decline than by either axis alone.

## Implications for diagnostics and therapeutics

The framework has substantial implications for microbiota-based diagnostics and therapeutics (Figure 3). If functional decline within retained taxa contributes to disease, composition-centered approaches may capture only part of the clinically relevant variation.

**Figure 3.**
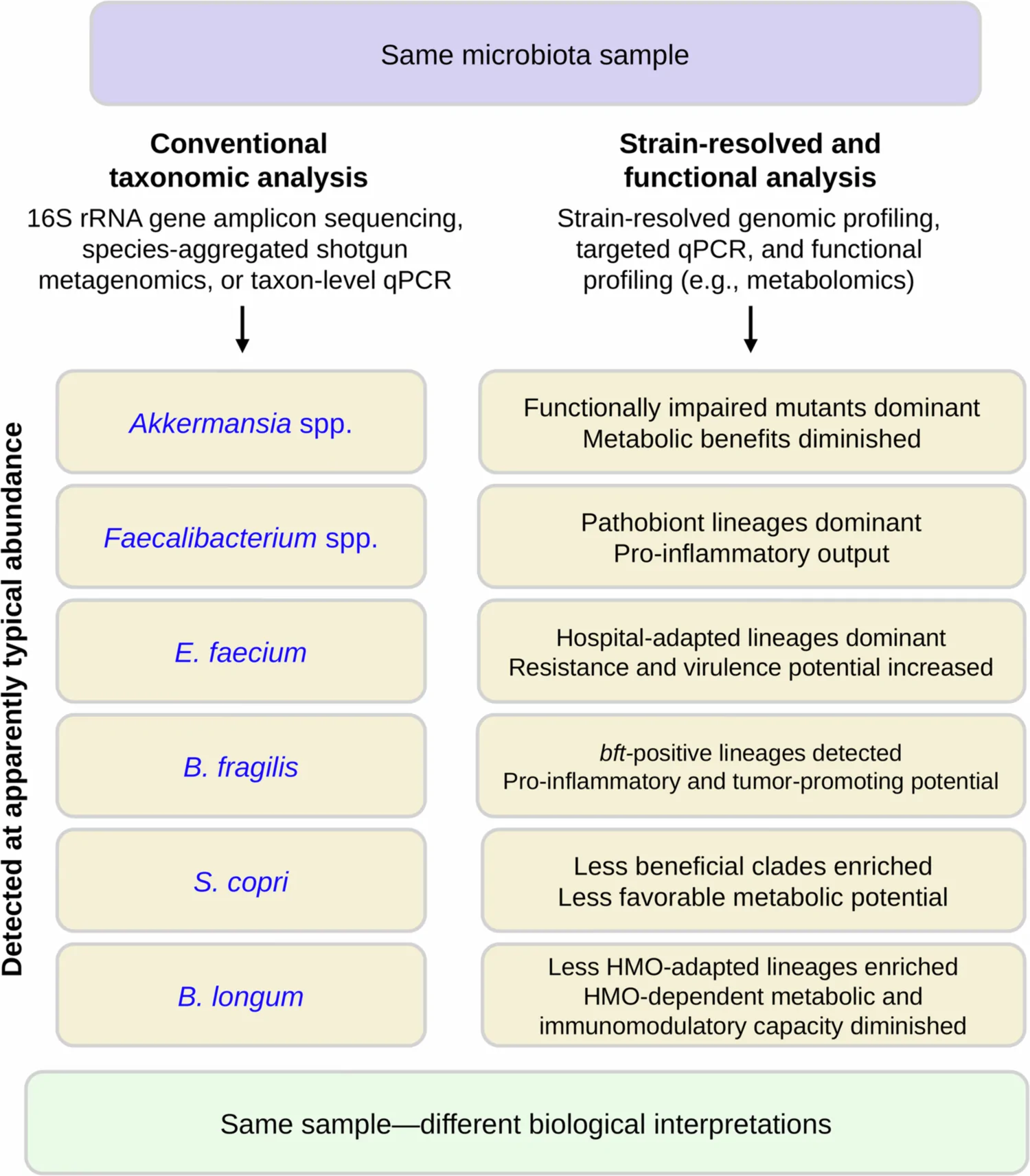
Diagnostic blind spot in conventional taxonomic microbiota analysis. Species-level and higher-level taxonomic profiles generated by 16S rRNA gene amplicon sequencing, species-aggregated metagenomics, or taxon-level qPCR assays may report *Akkermansia* spp., *Faecalibacterium* spp., *Enterococcus faecium*, *Bacteroides fragilis*, *Segatella copri*, and *Bifidobacterium longum* as present or at apparently typical abundance levels. In the same sample, strain-resolved genomic profiling and targeted variant assays may reveal functionally impaired or harmful populations, including metabolically compromised *Akkermansia* variants, pro-inflammatory *Faecalibacterium* lineages, hospital-adapted *E. faecium*, *bft*-positive *B. fragilis*, less beneficial *S. copri* clades, and less HMO-adapted *B. longum* lineages. Functional measurements, such as metabolomics, may further reveal the altered metabolic or immunomodulatory outputs of these populations. The same microbiota can therefore yield different biological and clinical interpretations depending on analytical resolution, illustrating how conventional taxonomic approaches may underestimate microbiota deterioration by overlooking functional deterioration within retained taxa. The figure is a schematic illustration of this conceptual diagnostic contrast and does not represent the output of an actual paired empirical analysis.

On the diagnostic side, species-level or higher-rank taxonomic profiles generated by 16S rRNA gene amplicon sequencing or species-aggregated shotgun metagenomics are often insufficient to detect functional decline (Figure 3). By grouping functionally distinct, or even opposing, strains and lineages within the same nominal taxon, these approaches may dilute signals of host-beneficial or harmful function and make them difficult to detect. Although targeted qPCR can distinguish specific variants when marker loci are known, most routine assays still measure taxon presence or abundance rather than functional state. They may therefore miss whether retained taxa are dominated by host-beneficial or pathobiont-like variants—for example, metabolically impaired *Akkermansia* variants, disease-associated *Faecalibacterium* lineages, or *bft*-positive *B. fragilis*. This limitation likely extends to other taxa in which clinically relevant functions vary below the resolution of standard taxonomic profiling.

Detecting functional decline requires strain- or lineage-resolved and function-oriented approaches, including high-resolution genomic profiling, variant-specific targeted assays, and functional readouts such as metabolomics (Figure 3). Although these approaches are increasingly feasible, cost, standardization, causal interpretation, and clinical validation remain major barriers.^80^ ^,^ ^81^

This framework shifts the central diagnostic question from taxonomic presence or abundance alone to functional state: not simply “Is *Akkermansia* present?” but “Which variants are present, and what do they do?”; not simply “Is *Faecalibacterium* abundant?” but “Which lineages dominate?”; and not simply “Is *B. fragilis* detected?” but “Does it carry *bft*?” Developing scalable assays, including targeted panels of functional decline-associated genomic and metabolic markers, is therefore a key near-term priority.

The therapeutic implications are equally important. Restoring diversity or reintroducing nominally beneficial taxa may be insufficient if the organisms introduced or enriched are functionally compromised. Donor-derived microbiota evaluated primarily at broad taxonomic resolution could provide limited benefit—or introduce risk—if such preparations contain ineffective or harmful variants. Likewise, live biotherapeutic products (LBPs) should not be selected on species identity alone. Therapeutic development should therefore include strain-level functional validation of administered organisms.

A central therapeutic question is whether functionally declined microbiota states are reversible. Across taxa, the key issue is whether strain replacement alone is sufficient, or whether the ecological conditions that favored declined variants must also be corrected. If an altered gut environment favors pathobionts or lineages with reduced host-beneficial capacity, durable reversal will likely require correcting that environment; otherwise, beneficial strains may fail to engraft or may again be outcompeted. Resolving this issue will help determine, in each case, whether effective intervention requires strain replacement, ecological correction, or both.

This framework also reframes dietary and antibiotic interventions as ecological pressures capable of reshaping lineage composition. If low-fiber diets favor pathobiont expansion, increasing fiber intake may help restore a more beneficial lineage balance. Antibiotic stewardship should likewise consider not only taxonomic loss, but also selection for and enrichment of functionally impaired survivors.

## Conclusion

Microbiota deterioration in industrialized populations has at least two dimensions, although research and clinical practice have focused largely on one: taxonomic loss. The disappearing microbiota framework has foregrounded this dimension, but taxonomic loss alone does not fully capture microbiota dysfunction. Functional decline within retained taxa provides a complementary axis that may help explain microbiota decline under modern industrialization-associated pressures. Because functional decline can remain undetected by standard taxonomic analyzes, the central question is not only whether key microbes are present, but which variants or lineages dominate and what functions they perform.

If taxonomic loss and functionally declined microbial states persist or accumulate within and across generations, microbiota restoration may become increasingly difficult, strengthening the rationale for early intervention. Clinical translation will require diagnostics that distinguish host-beneficial from functionally impaired or harmful populations, and therapies that restore microbial function rather than taxonomic composition alone. More broadly, the field must move toward function-first approaches to measuring, interpreting, and restoring the microbiota.

## Data Availability

Not applicable.
